# Measuring Safety Risks in Online Drug Sales: Empirical Study Based on Complex Network Theory

**DOI:** 10.2196/86876

**Published:** 2026-07-07

**Authors:** Rong Jiang, Bingke Xing, Lixin Shu

**Affiliations:** 1 Faculty of Pharmacy Naval Medical University Shanghai China; 2 School of International Pharmaceutical Business China Pharmaceutical University Nanjing China

**Keywords:** online drug sales, drug safety risks, risk measurement, complex network, complex network theory

## Abstract

**Background:**

With the rapid development of pharmaceutical e-commerce, online drug sales have led to additional safety risks, while providing convenient services to the public. These risks pose challenges to public health security and regulatory systems.

**Objective:**

This study aimed to quantitatively measure the safety risks of online drug sales.

**Methods:**

We collected data on cases of violation in online drug sales from government websites and established a complex network model consisting of 116 nodes and 152 edges for the safety risks in online drug sales based on complex network theory. We explored network topological properties through complex network characteristic indicators (eg, average path length, diameter, degree, and degree distribution). In addition, we followed the Technique for Order Preference by Similarity to Ideal Solution (TOPSIS) method to identify key risk factors in online drug sales safety, calculate the relative nearness degree value of each risk node, and rank them accordingly.

**Results:**

Data on 458 cases of violation were collected. The key risk nodes in the complex network model for safety risks in online drug sales were identified as the failure to (1) strictly perform prescription review and dispensing duties, (2) issue prescriptions through internet hospitals, (3) establish drug procurement and acceptance records, and (4) establish drug sales records, in addition to (5) consumers failing to provide prescriptions.

**Conclusions:**

Based on the risk measurement results, regulatory authorities can formulate targeted risk prevention and control measures for key risks, such as failure to strictly perform prescription review and dispensing duties and consumers failing to provide prescriptions. By building a tighter and stronger regulatory network for online drug sales and improving regulatory efficiency, this approach could maintain order in the online drug sales market and enhance the overall level of public health security.

## Introduction

In recent years, with the rapid development of internet technology, the medical e-commerce industry has surged to prominence. The online drug sales market has entered a period of rapid development, emerging as a new business model within the pharmaceutical sector. Compared with traditional drug-purchasing methods, online drug sales in China allow consumers to purchase drugs online through various online shopping platforms, such as JD.com, Taobao, and Meituan, breaking the time and space constraints of traditional pharmacies [1]. In the first half of 2025, China’s online pharmacy market recorded pharmaceutical sales exceeding 40 billion yuan (US $6 billion), representing a year-on-year increase of 20.36% [2].

However, compared to offline channels, the virtual nature, anonymity, and cross-regional reach of online sales make it a high-risk channel for circulating counterfeit and substandard drugs and illegal sales worldwide [3,4]. China emphasizes the development of online drug sales, having successively revised and introduced a series of policies to strengthen risk management concepts and enhance systematic governance. In this study, safety risks associated with online drug sales referred to various potential factors or hazards that may lead to issues regarding drug quality and consumer drug safety during pharmaceutical transactions conducted via online platforms. In the literature on safety risks in online drug sales, researchers have used both qualitative and quantitative analytical methods to identify these risks. Ahmed et al [5] reported that online drug sales carry risks, such as drug abuse, inconsistent quality, misleading product claims, and difficulties in protecting consumer rights. Xu and Wang [6], Huang et al [7], and Xie [8] found that the safety risks associated with online drug sales in China include imperfect traceability mechanisms for online drug sales, inadequate logistics and distribution systems, nonstandardized prescription management, insufficient platform oversight, incomplete laws and regulations, and a lack of online pharmaceutical service systems. Some scholars have used relevant theories or models to study the safety risks associated with online drug sales, exploring the causes or effects of these risks. Liu [9] used risk management theory and found that the inadequate regulatory system, weak internal and external constraints within enterprises, and multiple game dynamics in the market environment are the key factors leading to safety risks in online drug sales. Hu et al [10] used failure mode and effects analysis to assess potential safety risks associated with purchasing prescription drugs from online pharmacies, with results indicating that the reuse of paper prescriptions poses the highest risk. Oriakhi et al [11] conducted a cross-sectional study of online pharmacies during the COVID-19 pandemic and found that 47% were “rogue” pharmacies; furthermore, most legitimate pharmacies relied on online consultations instead of prescription reviews, highlighting the serious patient safety risks posed by the lack of safety checks and inadequate regulation.

In recent years, quantitative analysis methods based on case texts have gradually been applied to the field of safety risk research. Zhang et al [12] established a risk evaluation index system based on bus fire accident cases, identifying the primary factors contributing to these incidents. Pathan et al [13] used analytical hierarchy and Technique for Order Preference by Similarity to Ideal Solution (TOPSIS) methods to assess flood risk in the city of Navsari, Gujarat, India, identifying vulnerable areas more susceptible to inundation during floods. Shen et al [14] collected investigation reports on construction safety accidents in rural self-built houses from 2014 to 2022 and established a Bayesian network model to elucidate the key causes of these accidents. These findings demonstrate that case-based quantitative analysis methods have matured, but their application in research on the safety risks in online drug sales remains unexplored.

For the past few years, complex network theory, by virtue of its strong advantages in quantitative and visual analysis, has been widely applied in safety analysis across various fields, such as transportation [15], construction [16], coal mining [17], and power systems [18]. Based on this theory, researchers primarily extract risks from case texts, establish correlations between risks, and measure risks using complex network statistical metrics. Wang et al [19] extracted relationships between hazards in Beijing subway accident cases and constructed a network diagram, identifying key hazards using degree, clustering coefficient, and betweenness centrality. However, network indicators, such as degree and betweenness centrality, only reflect risk characteristics from different dimensions and cannot comprehensively measure the importance of risks on their own. Therefore, some scholars use the TOPSIS method to synthesize multiple complex network statistical indicators into a single metric for assessing the extent to which risk affects security. The TOPSIS method is suitable for scenarios involving the ranking and selection of multiple indicators and evaluation objects. By calculating the proximity to the ideal solution, it enables the objective integration of multidimensional indicators, thereby avoiding the one-sidedness associated with a single evaluation dimension. Zhang et al [20] applied the entropy weight–TOPSIS method and evaluation metrics, including degree centrality, betweenness centrality, and PageRank value, to assess the importance of risk nodes in severe traffic accidents in Sichuan Province, China. Online drug sales involve multiple entities and processes, containing numerous safety risk factors that influence each other. Therefore, applying complex network theory to construct the relational structure among safety risk factors of online drug sales, and using the TOPSIS method to integrate multiple network indicators, can scientifically measure risk and has good applicability in research on safety risks in online drug sales.

Currently, China has established a rigorous regulatory framework for online drug sales centered on risk management. Regulatory authorities have invested substantial resources in inspections and enforcement, generating a vast and systematically rich body of publicly available administrative penalty cases. These cases provide a solid data foundation for conducting a reverse analysis of the safety risks in online drug sales models from a risk perspective. However, no existing research has systematically mined and quantitatively analyzed these cases. Therefore, this study aimed to fill this research gap. By collecting cases of violation, we innovatively applied complex network theory to construct a complex network model for safety risks in online drug sales and identified key risks using the TOPSIS method based on network statistical indicators, filling a research gap. The findings will provide evidence-based support for regulators to implement precise governance and optimize resource allocation, while also offering methodological references for risk research and practice in similar markets worldwide.

## Methods

### Data Collection

We collected cases of violation in online drug sales from January 1, 2014, to June 30, 2024. Data sources included the Peking University Law Database, China Judgments Online, the National Medical Products Administration (NMPA) website, provincial/municipal medical product administration websites, and administrative punishment announcements from market supervision bureaus. The following search terms were used: “internet drug” (互联网药品) OR “online drug sales” (药品网络销售) OR “online pharmacy” (网上药店).

A total of 772 violation online drug sales cases were retrieved. After removing duplicates, 648 (83.94%) cases remained. Next, we conducted a detailed review of the cases and further excluded some based on the following exclusion criteria: (1) cases violation unrelated to online drug sales, such as online sales of medical devices, cosmetics, health supplements, or offline physical pharmacy operations; (2) cases where the information was too brief to identify a risk transmission chain; and/or (3) cases with extensive content but vague descriptions of illegal acts, using generalizations instead of process details, making it difficult to determine risks and their interrelationships. Finally, 458 (70.68%) cases of violation in online drug sales were obtained.

Two researchers jointly reviewed the facts, processes, and legal grounds for penalties in the 458 cases, identifying specific risk points for each case. Through discussion, similar items were consolidated and duplicates removed to obtain a unique set of risk factors. Subsequently, the two researchers independently grouped the risk factors based on semantic similarity. After resolving any disagreements through discussion, the factors were preliminarily categorized into several risk categories, and three experts were invited to review and confirm the classification results. Adjustments were made based on the experts’ feedback, resulting in a final risk list and risk categories.

### Complex Network Theory

Complex network theory commonly uses network diagrams to study system structures and the interrelationships between structure and system function. The key to complex network modeling lies in defining nodes and edges [21]. Based on the fundamental knowledge of complex network theory and the characteristics of online drug sales, we made the following assumptions:

- Assumption 1: The nodes in the model represent risks, and the edges represent the interrelationships between the risks. In the safety risk network model of online drug sales, a node activation indicates that the corresponding risk is active, and this node’s activation may directly trigger violation incidents. However, in most cases, violations result from the intertwined and combined effects of multiple risks. Therefore, the interrelationships between risks can be abstracted as edges within the safety risk network of online drug sales.
- Assumption 2: The model is a directed network, with directions representing the flow of risk transmission. Violations in online drug sales are not caused by a single risk factor but result from the combined effects of multiple interconnected risks. These risks are not isolated but interact with one another. Based on the complete facts of the violations in the penalty document, the sequence of events and the logical causal relationships leading to the risks are identified, and the corresponding links between preceding and subsequent risks are elucidated. Risks with clear triggering or evolutionary relationships are represented as directed edges in a complex network to illustrate the transmission relationships between risks. The risk transmission chain clearly illustrates the progression of cases of violation and precisely reveals the interconnections and chain reactions among risks. Therefore, we simplified violations in online drug sales into a directed network to represent the transmission relationships.

Based on these two assumptions, risk transmission chain data are converted into an adjacency matrix. Using Gephi software, the number of times connections occur between risks is used as weighting to form a complex network composed of multiple risk transmission chains. Analyzing the network’s indicator parameters can reveal its structural characteristics and evolutionary patterns. Common indicators include average path length, diameter, degree, betweenness centrality, eigenvector centrality, and clustering coefficient [22].

#### Average Path Length and Diameter

The average path length refers to the mean value of all the shortest paths between any two risk nodes, reflecting the strength of mutual influence between factors [23]. It serves as a crucial metric for assessing risk propagation efficiency and connectivity. The network diameter denotes the maximum distance between any two nodes in the network, where the distance between directly connected nodes is 1.

#### Degree

Degree is one of the key topological properties in complex networks, representing the number of connections a node has with other nodes and reflecting the node’s direct effect on the network [24]. A higher degree indicates that the node has more extensive direct connections with other nodes in the network, potentially making it a critical node. For directed networks, degree centrality includes in-degree, out-degree, and total degree.

#### Betweenness Centrality

In complex networks, the betweenness centrality measures a node’s connectivity by quantifying the ratio of the number of shortest paths passing through it to the total number of shortest paths in the network [25]. A higher betweenness centrality indicates that a significant number of shortest paths traverse that node. Consequently, the risks represented by the node play a more pivotal role as intermediaries within the risk network, substantially affecting risk propagation across the entire network.

#### Eigenvector Centrality

Eigenvector centrality is a metric for measuring node importance, considering not only the number of nodes directly connected to it but also the effect of its adjacent nodes [26]. The higher the eigenvector centrality, the more connections a node has with core nodes, indicating a stronger radiating effect and influence within the network.

#### Clustering Coefficient

The clustering coefficient measures the clustering properties of nodes within a network [27]. It is the ratio of the actual number of edges connecting a node to its neighbors to the maximum possible number of edges, with values ranging from 0 to 1. This metric intuitively reflects the closeness of connections surrounding a node, indicating whether its neighboring nodes are interconnected.

### Risk Measurement

Different metrics in complex networks analyze the effect of nodes from various perspectives. Relying solely on a single metric makes it difficult to fully measure the impact of nodes within a network [28]. To integrate multiple attribute metrics of a single node and thereby assess the effect of individual risks on the final outcome, we used the TOPSIS method to calculate the risk nearness degree C_i_. The TOPSIS method is a classical multiattribute decision-making approach that comprehensively considers multiple attribute indicators, avoiding the one-sidedness of single-criterion decision-making. It is widely applied in multiobjective decision-making [29,30] and risk measurement studies [31,32]. The steps in calculation are detailed in Multimedia Appendix 1.

The fundamental approach of the TOPSIS method for risk measurement is as follows: Each risk node within the complex network model for safety risks in online drug sales is treated as a decision scheme in TOPSIS. Four metrics (degree, betweenness centrality, eigenvector centrality, and clustering coefficient) represent the four attributes of each decision scheme. A decision matrix based on the attribute values of each indicator was construct, the distance between each alternative and the ideal solution was measured, and their nearness degree was calculated. The nodes were ranked according to their nearness degree, where a higher degree indicated greater proximity to the positive ideal solution and a stronger influence.

### Ethical Considerations

This study did not involve any research on human or animal participants.

## Results

### Basic Information About Cases of Violation

**Figure 1:**
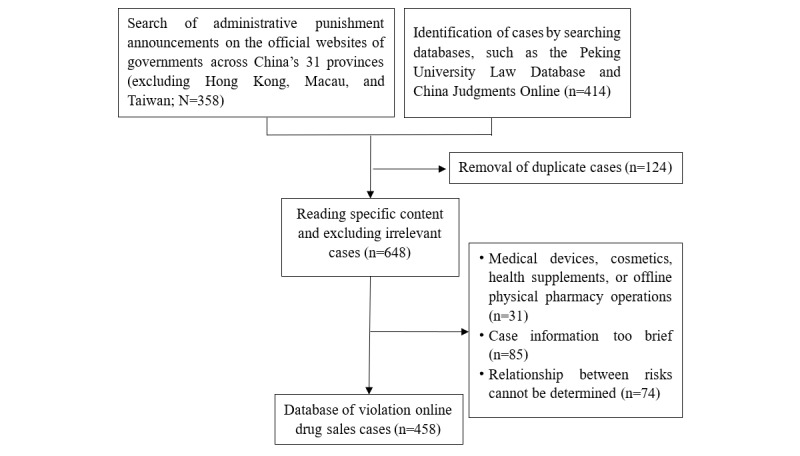
Flowchart for selecting cases of violation related to online drug sales.

The case-screening process is shown in Figure 1. We collected a total of 458 cases of violation in online drug sales (Table 1). Over time, the overall number of cases showed an upward trend, accelerating significantly after 2018. This indicates that as internet penetration and e-commerce platforms develop, the scale of online drug sales continues to expand, and illegal incidents gradually increase. Cases of violation primarily occurred in economically developed or densely populated regions, such as Zhejiang Province, Guangdong Province, Fujian Province, and Beijing Municipality. These areas feature high internet penetration rates and substantial pharmaceutical market demand, making them potential hotspots for violations. The entities that commit violations include not only pharmaceutical trading enterprises (eg, retail pharmacies and wholesale drug distributors) and related individuals (eg, licensed pharmacists) but also enterprises that do not originally engage directly in drug trading, including technology companies, trading companies, and food stores. This underscores the need for enhanced oversight, not only of pharmaceutical businesses and individuals, but also of diverse entities operating in the field of online drug sales.

**Table 1:** Basic information about cases of violation in online drug sales (N=458).

Item		Cases, n (%)
**Penalty year**		
	First half of 2024	62 (13.54)
	2023	157 (34.28)
	2022	76 (16.59)
	2021	58 (12.66)
	2020	42 (9.17)
	2019	26 (5.68)
	2018	13 (2.84)
	2017	5 (1.09)
	2016	1 (0.22)
	2015	3 (0.66)
	2014	4 (0.87)
	Not reported	11 (2.40)
**Region**		
	Zhejiang Province	103 (22.49)
	Guangdong Province	63 (13.76)
	Fujian Province	38 (8.30)
	Beijing Municipality	27 (5.90)
	Tianjin Municipality	26 (5.68)
	Sichuan Province	21 (4.59)
	Other	180 (39.30)
**Illegal subject**		
	Pharmaceutical trading company	267 (58.30)
	Individual	67 (14.63)
	Technology company	27 (5.90)
	Trading company	25 (5.46)
	Food stores	14 (3.06)
	Other	58 (12.66)

### Network Construction Process

#### Identify Risks

The 458 cases of violation represented actual security incidents that occurred or potential security risks that existed, ultimately leading to the imposition of penalties. Drawing upon these cases of violation as the source material, we identified potential risk points within the cases, extracted 116 risk factors, and categorized them into 8 aspects: business qualifications, 16 (13.79%) risks; drug procurement, 14 (12.07%) risks; transportation and delivery, 9 (7.76%) risks; information display, 11 (9.48%) risks; advertising and promotion, 17 (14.65%) risks; prescription management, 19 (16.38%) risks; pharmaceutical services, 3 (2.59%) risks; and drug sales, 27 (23.28%) risks. Further details are provided in Multimedia Appendix 2.

#### Identify Risk Transmission Chains

Compared to the traditional drug sales model, online sales involve multiple entities, including e-commerce platforms, online pharmacies, internet hospitals, consumers, and regulatory authorities, as well as multiple operational stages, such as business licensing, information display, prescription verification, drug delivery, and pharmaceutical services. These factors result in complex coupled transmission relationships among risks. Furthermore, when collating cases of violation in online drug sales, violations sometimes result not from a single risk but also from the coupling or derivation of multiple risks under certain conditions. Safety risks online drug sales possess a certain dynamic propagation characteristic.

We extracted and analyzed information from cases of violation in online drug sales, using risks as nodes and arrows to represent the transformation and evolution relationships between risks, thereby forming a chain of safety risk transmission in online drug sales. A total of 509 risk transmission chains were constructed. Taking the case of a tobacco and liquor retailer in Ningbo City as an example, Textbox 1 illustrates the process of extracting the risk transmission chain from facts about the violation.

**Example of extracting the safety risk transmission chain in online drug sales.**

Case source:

Administrative Penalty Information Public Disclosure, Zhejiang Province

Facts about the violation:

Investigation revealed that in July 2021, the involved party, Ningbo *** Tobacco & Alcohol Store, purchased one box (100 pieces) of Yunnan Baiyao Plasters (drug approval number: GYZZ Z20073016) at a price of 17 yuan/box (US $2.51/box), or 0.17 yuan/piece (US $0.02/piece) from the Ningbo Jiangdong Modern Department Store Market but did not retain the purchase invoice. The involved party could not recall the specific stall number. The involved party commenced business on August 5, 2021, primarily engaged in the sales of prepackaged food, daily necessities, and tobacco products, with an operating area of ~80 m^2^. Until the case was identified, they had sold a total of 70 pieces of Yunnan Baiyao Plasters at a price of 0.5 yuan/piece (US $0.07/piece). Furthermore, in September 2021, they established a Tmall Shop online store on the Ele.me platform, primarily for retail general merchandise and the sale of Yunnan Baiyao Plasters. In October 2021, they began listing and selling Yunnan Baiyao Plasters on Tmall Shop. The product page contained the text “Yunnan Baiyao Plaster 1 piece ¥0.5”. When the text was clicked, the page displayed content and images including “Yunnan Baiyao Plaster OTC [Hemostasis/ Analgesia/ Anti-inflammatory/ Wound Healing/ Lightweight & Breathable],” “Add to Cart,” “¥20 minimum for delivery,” “7-day no-reason returns supported”, etc. Until the case was identified, the online store had sold a total of 15 pieces of Yunnan Baiyao Plasters at a price of 0.5 yuan/piece (US $0.07/piece).

Risk transmission chain:

Failure to obtain a drug business license (A01) → procuring drugs from unqualified individuals or enterprises (B01) → inability to provide drug procurement vouchers (B11) → failure to establish drug procurement and acceptance records (B12) → illegal sale of over-the-counter (OTC) drugs (H08)

#### Construct Complex Network Models

**Figure 2:**
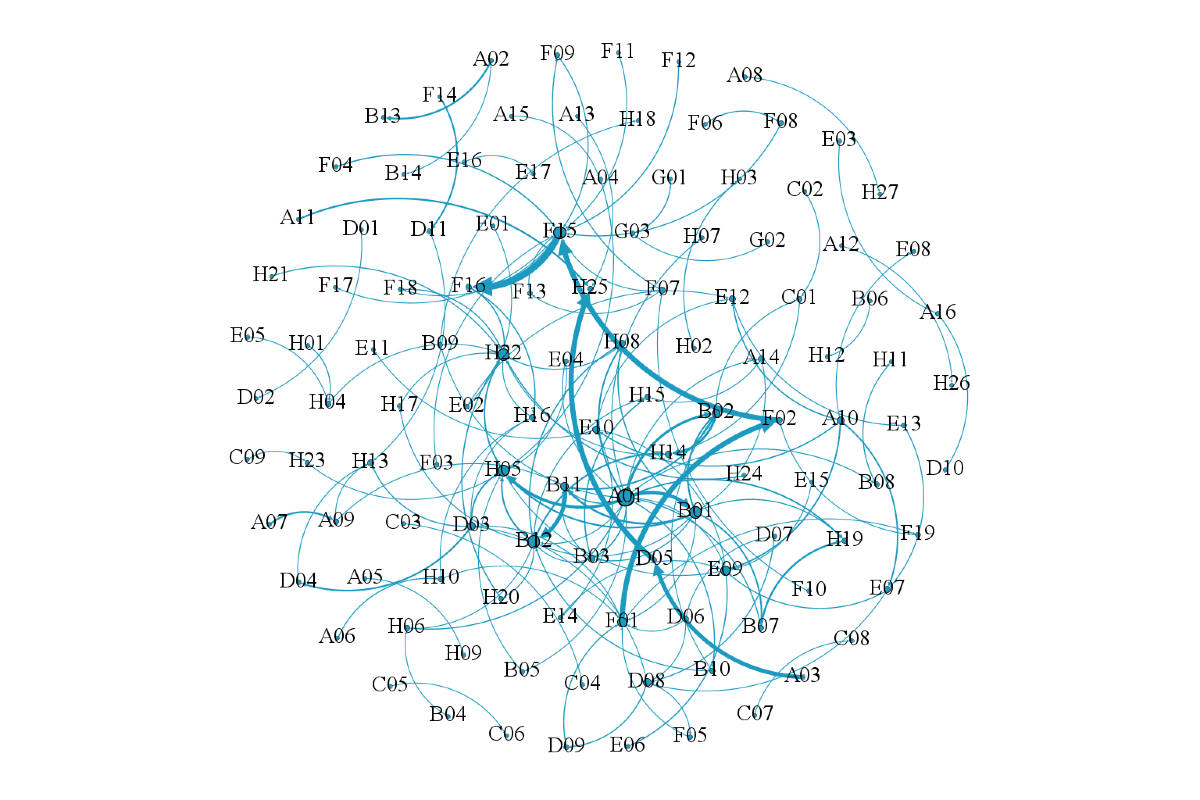
Complex network model for safety risks in online drug sales.

Based on the aforementioned assumptions and risk transmission chains, we used the social network analysis software Gephi 0.10.1 to construct a complex network model for safety risks in online drug sales. The network consisted of 116 nodes and 152 directed weighted edges. Figure 2 shows the visualization result.

### Analysis of Network Topology Characteristics

#### Average Path Length and Diameter

The average path length of the complex network model for safety risks in online drug sales was 2.453, indicating that it takes 2-3 steps to establish a connection between two risk points (relatively small average separation between risk nodes). This implies that in the network model, if a risk occurs at one node, it can be transmitted to other nodes over a short distance, quickly forming a chain reaction that impacts the safety of the entire online drug sales system.

The network diameter of the network model was 8, indicating that it takes a maximum of 8 steps for a risk node to affect another risk node within the network. Therefore, a risk transmission chain can be established within 0-8 steps.

#### Degree and Degree Distribution

**Figure 3:**
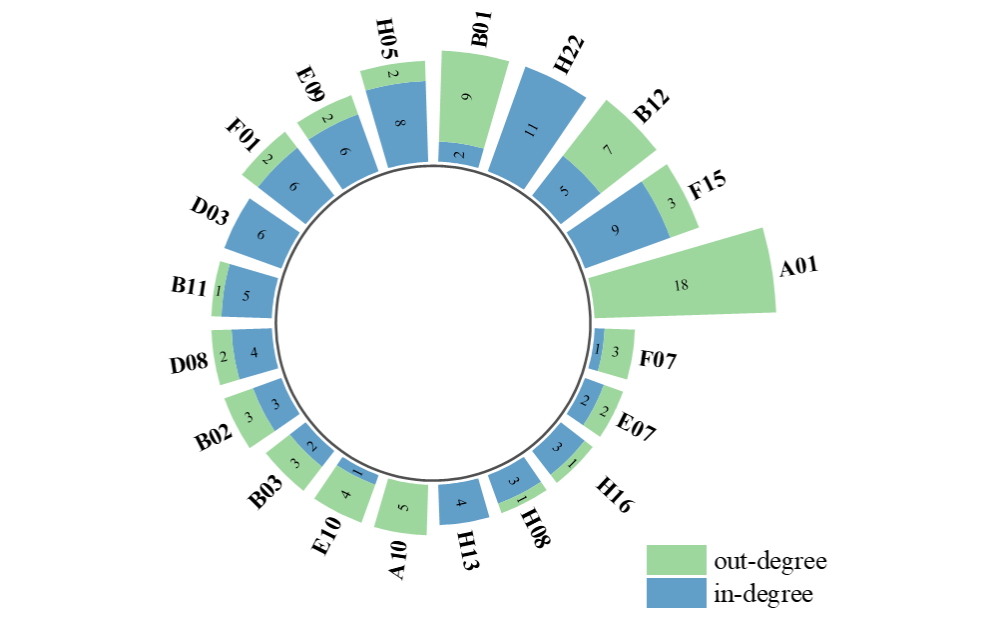
Top 20 risk nodes by degree in the complex network model of safety risks in online drug sales.

Figure 3 lists the top 20 risk nodes, arranging all nodes in descending order of degree value. The average degree of the complex network model was 2.621, indicating that each node in this network model is connected to an average of 2.621 other nodes. This means that if one risk changes, it can, on average, affect 2-3 other related risks.

Regarding out-degrees, A01 (failure to obtain a drug business license) had the highest out-degree value of 18. This indicates the risk of failing to obtain a drug business license can propagate to 18 risk nodes within the network, with multiple risk evolution pathways. Regarding in-degrees, H22 (failure to establish drug sales records) had the highest value of 11. This indicates that failure to establish drug sales records can evolve from 11 other risks, making it relatively susceptible to influence from other risks.

**Figure 4:**
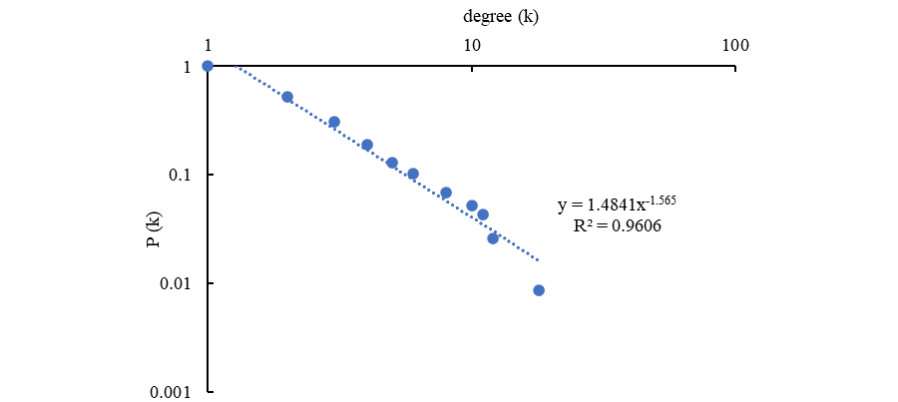
Cumulative degree distribution of nodes in the complex network for safety risks in online drug sales.

The cumulative degree distribution of nodes in the complex network model is shown in Figure 4. The cumulative degree followed a power-law distribution, with the fitting result being P(k)= ~1.4841k^–1.565^ and excellent fitting quality (R²=0.9606). This indicates that the complex network model established in this paper exhibits scale-free properties, with most nodes having low degrees and only a small number possessing high degrees. Risks in networks tend to concentrate around core nodes, and the scale-free characteristic endows the network with robustness against random failures and vulnerability to targeted attacks [33]. Therefore, targeted attacks should be launched against high-risk nodes with large degrees within the network to disrupt their risk transmission pathways. This will increase the diameter and average path length of the complex network model, thereby slowing the evolution and spread of risks.

#### Betweenness Centrality

**Figure 5:**
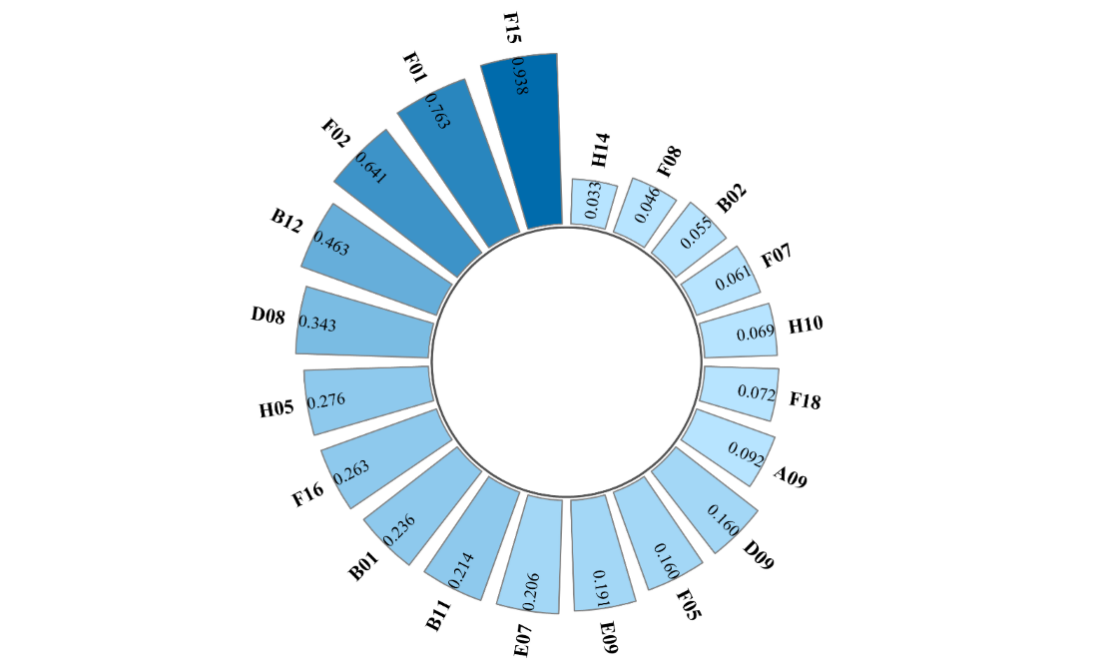
Top 20 risk nodes by betweenness centrality in the complex network model for safety risks in online drug sales.

Figure 5 lists the top 20 risk nodes, ranking all risk nodes in descending order of betweenness centrality. F15 (failure to strictly perform prescription review and dispensing duties) exhibited the highest betweenness centrality at 0.938, followed by F01 (consumers failing to provide prescriptions) and F02 (failure to issue prescriptions through internet hospitals), with betweenness centralities of 0.763 and 0.641 respectively. This indicates that within the complex network model, these three nodes lie along the shortest paths connecting numerous other node pairs, serving as critical bridges in risk transmission. For instance, F15 (failure to strictly perform prescription review and dispensing duties) acts as a pivotal node linking risks such as a consumer uploading a blank prescription form and the sale of prescription drugs without prescriptions. A risk represented by a node with high betweenness centrality often triggers further risks along transmission pathways. Therefore, establishing specialized risk management plans for these critical nodes can effectively slow the rate of risk evolution and propagation, mitigating and preventing the chain reaction of multiple risk events.

**Figure 6:**
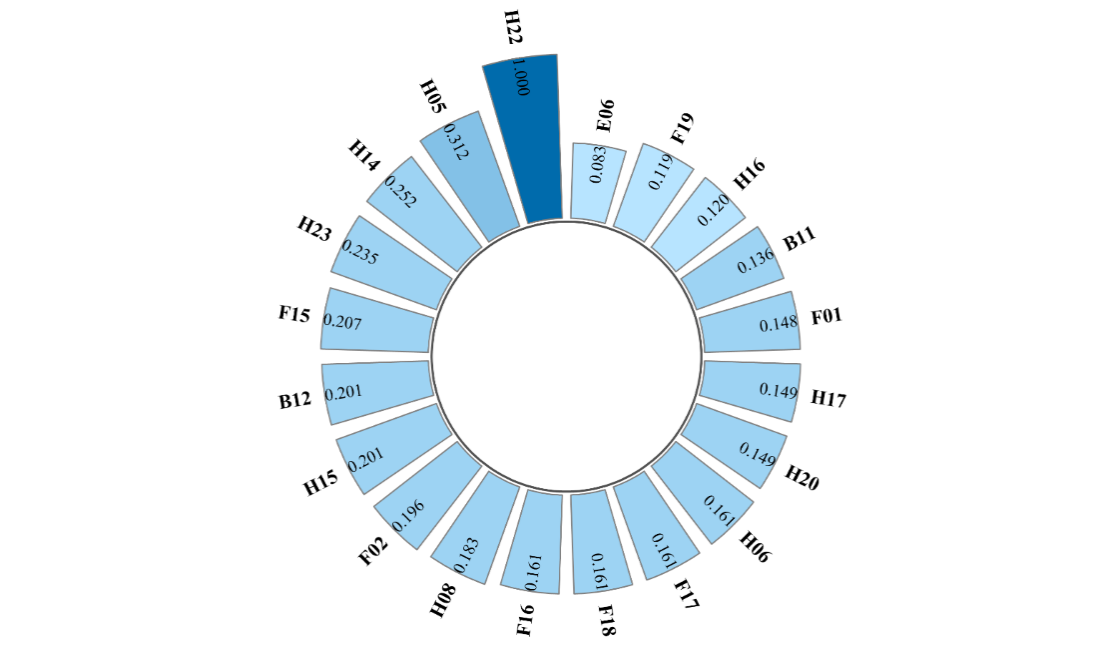
Top 20 risk nodes by eigenvector centrality in the complex network model for safety risks in online drug sales.

Figure 6 lists the top 20 risk nodes, ranking all nodes in descending order of eigenvector centrality. H22 (failure to establish drug sales records) exhibited the highest eigenvector centrality at 1.00, indicating that failure to establish drug sales records is linked to multiple critical risk nodes. Among the risk nodes with elevated eigenvector centrality, various drug sales–related risk nodes were encompassed, such as H05 (illegal drug sales), H14 (selling imported drugs without drug approval documents), and H23 (drug sales records are not authentic or complete). This implies that the definition of eigenvector centrality aligns with our practical understanding of safety in online drug sales. Specifically, most risks in the sales segments are typically linked to key risk nodes in the complex network model. The combined influence of these risk nodes increases the probability of sales risks occurring.

#### Clustering Coefficient

**Figure 7:**
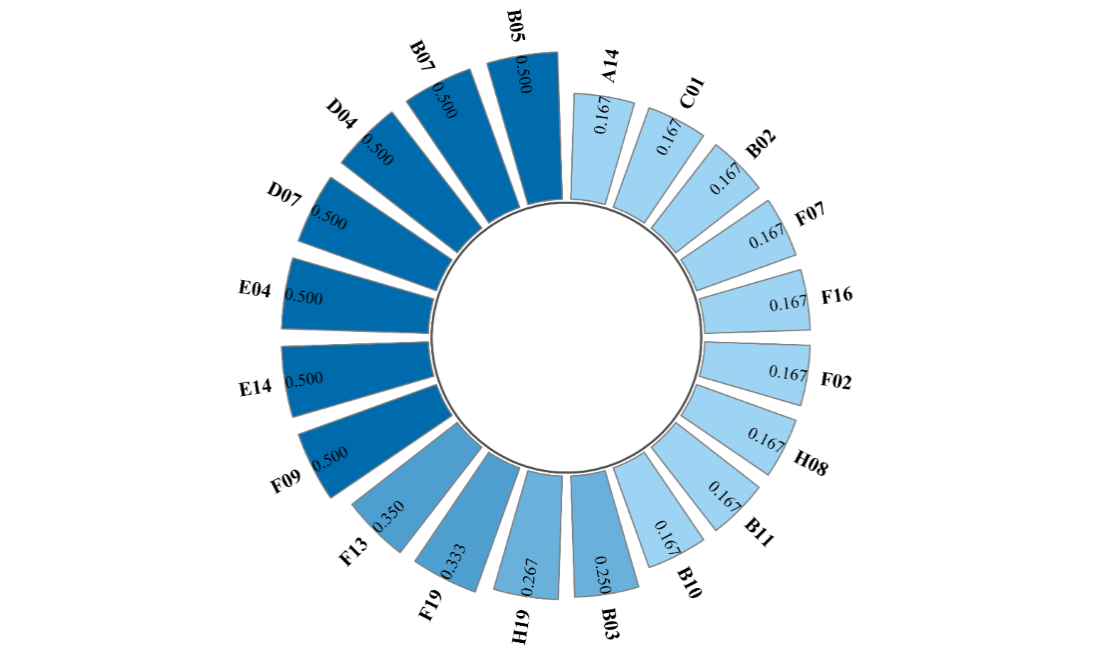
Top 20 risk nodes by clustering coefficient in the complex network model for safety risks in online drug sales.

Figure 7 lists the top 20 risk nodes, ranking all nodes in descending order of clustering coefficient. Ten risks nodes, including B05 (using others’ information to buy drugs from various hospitals), B07 (procuring counterfeit drugs), and D04 (failing to display qualification information of pharmacists or other pharmaceutical technical personnel) among others, exhibited the highest clustering coefficient of 0.50. This indicates a clustering effect among adjacent risk nodes for these risks. For example, when someone uses another person’s information to purchase drugs at hospitals across different regions, the strong correlation between adjacent risks in the network, such as A01 (failure to obtain a drug business license) and H05 (illegal drug sales), may trigger a chain reaction within a localized area.

The average clustering coefficient of the complex network model was calculated to be 0.077. Using Gephi 0.10.1 software to generate a random network with the same number of nodes and edges yielded an average clustering coefficient of 0.008. Therefore, compared to a random network of the same scale, the complex network model exhibits a higher average clustering coefficient. This indicates the presence of strong local clustering structures within the network, where risks tend to concentrate more readily in specific segments.

### Comprehensive Ranking of Network Risks

The importance of risk nodes in the complex network for safety risks in online drug sales is influenced by multiple factors. Different indicators in complex network theory analyze the effect of nodes from different perspectives. Relying solely on a single indicator makes it difficult to fully measure the importance of nodes in the network [28]. Therefore, we adopted the TOPSIS method, effectively integrating four indicators from the complex network model (degree, betweenness centrality, eigenvector centrality, and clustering coefficient) to scientifically and reasonably measure the importance of each risk node in the network. Based on the TOPSIS calculation results, the risk nodes were ranked in descending order of Cᵢ; the top 15 nodes are shown in Table 2, while the complete ranking of all nodes is provided in Multimedia Appendix 3.

**Table 2:** Ranking of the top 15 risk nodes by C_i_ value for nodes in the complex network model for safety risks in online drug sales.

Node	C_i_ in descending order
F15	0.5408
F01	0.4638
F02	0.4419
B12	0.3956
H22	0.3872
F19	0.3561
H19	0.3547
B07	0.3518
E14	0.3477
F09	0.3477
F13	0.3477
B05	0.3471
D04	0.3471
D07	0.3471
E04	0.3471

In this paper, a higher relative nearness degree value for a node indicated that the node was more important in the complex network model for safety risks in online drug sales and represented a key node in the risk network. As shown in Table 2, the top-ranked risks in the complex network model included F15 (failure to strictly perform prescription review and dispensing duties), F01 (consumers failing to provide prescriptions), and F02 (failure to issue prescriptions through internet hospitals), with relative nearness degrees of 0.5408, 0.4638, and 0.4419, respectively. These risk nodes occupied critical positions within the network, and once manifested, they rapidly propagated to other risk nodes in the network. Among these, F15 (failure to strictly perform prescription review and dispensing duties) exhibited the highest relative nearness degree at 0.5408, making it the most critical risk factor in the complex network for safety risks in online drug sales that requires prioritized prevention and control.

## Discussion

### Principal Findings

With the rapid development of internet technology, significant changes have occurred in public drug-purchasing patterns. Online drug sales have gradually become a primary channel for the public to purchase drugs due to their convenience and efficiency [34]. However, this has also led to a series of issues and risks, such as the sale of prescription drugs without prescriptions and discrepancies between drug information displayed online and the actual products. Our study introduced complex network theory into the research on safety risks in online drug sales by constructing a network model to analyze risk propagation patterns and using the TOPSIS method to measure risks. The results showed that the key risk nodes in the model are F15 (failure to strictly perform prescription review and dispensing duties), F01 (consumers failing to provide prescriptions), F02 (failure to issue prescriptions through internet hospitals), B12 (failure to establish drug procurement and acceptance records), and H22 (failure to establish drug sales records).

We collected a total of 458 cases of violation. Based on the facts of violations and combined with legal and regulatory requirements, potential risk points in these cases were identified. Ultimately, 116 risks were extracted from the cases and categorized into 8 aspects: business qualifications, drug procurement, transportation and delivery, information display, advertising and promotion, prescription management, pharmaceutical services, and drug sales. Based on complex network theory, a complex network model for safety risks in online drug sales was established, comprising 116 nodes and 152 directed weighted edges.

Analysis of the topological properties of the complex network model revealed that A01 (failure to obtain a drug business license) has the highest total degree, with the maximum risk transmission or reception relationships. Although purchasing medicines from legal online pharmacies has been accepted in higher-income countries, the market has always been plagued by illegal suppliers, posing a significant risk to patient safety [35]. Burrell [36] analyzed the operational structure of illegal online pharmacies from the perspective of cross-border cybercrime, pointing out that it exhibits an adaptive strategy and high profit and low risk characteristics and is deeply intertwined with public health safety risks. Meanwhile, the network’s cumulative degree follows a power-law distribution, exhibiting scale-free characteristics. F15 (failure to strictly perform prescription review and dispensing duties) exhibits the highest betweenness centrality, serving as a crucial bridge in risk transmission. Consistent with our findings, Oriakhi et al [11] investigated the operation of online pharmacies and found that among the 116 online pharmacies evaluated, 47% were illegal, often selling prescription drugs without prescriptions or consultations. H22 (failure to establish drug sales records) has the highest eigenvector centrality, connecting to multiple important risk nodes in the network. Ten risk nodes, including B05 (using others’ information to buy drugs from various hospitals), B07 (procuring counterfeit drugs), and D04 (failure to display qualification information of pharmacists or other pharmaceutical technical personnel), have the highest clustering coefficients, indicating a clustering effect among adjacent risk nodes of these risks.

The TOPSIS method results indicate that in the complex network model, the top-ranked risks include F15 (failure to strictly perform prescription review and dispensing duties), F01 (consumers failing to provide prescriptions), F02 (failure to issue prescriptions through internet hospitals), B12 (failure to establish drug procurement and acceptance records), and H22 (failure to establish drug sales records). These risk nodes occupy crucial positions within the network and can rapidly propagate to other risk nodes. A study conducted in Saudi Arabia on the online purchase of antibiotics found that before selling antibiotics, up to 33.3% of online pharmacies do not require customers to provide a prescription [37]. The study further pointed out that 45% of these websites are fake or only sell local antibiotics [37]. Ahmed et al [38] conducted a systematic review on the issue of substandard and counterfeit drugs sold online, identifying challenges, such as the lack of knowledge and awareness among consumers and doctors, as well as the use of internet platforms and social media for direct-to-consumer advertising. Furthermore, Xiao [39] conducted a systematic analysis of the regulatory policies for online pharmacies in China and reported that the revision of the Drug Administration Law in 2019 was the first national-level legal framework for regulating online pharmacies in China. However, as artificial intelligence (AI)–driven online pharmacies are rapidly emerging, issues such as the responsibility boundaries of online platforms and the legal status of AI doctors and pharmacists still face legal and ethical controversies. These new business models will further exacerbate the complexity of safety risks in online drug sales. Digital health interventions, training programs for medical service providers, and public awareness campaigns tailored to specific situations are key approaches to bridging the gap between cognition and practice [40]. To mitigate the aforementioned risks, regulatory authorities should popularize the knowledge of prescription drugs to the public through various channels and media, including definitions, online purchasing procedures, and usage risks, thereby guiding the public to voluntarily purchase prescription drugs with prescriptions. Online pharmacies engaged in prescription drug sales should regularly organize licensed pharmacists to participate in relevant training or continuing education courses to enhance their sense of responsibility and professional competence. Furthermore, standardized and unified electronic prescription review processes should be established to ensure the professionalism and compliance of online prescription reviews. In addition, regulatory authorities should conduct regular inspections of online drug operating enterprises, focusing on whether they have established complete and accurate drug procurement, acceptance, and sales records, as required, and whether these records enable drug traceability.

### Strengths and Limitations

This study quantitatively measured risks in online drug sales and identified key risks, providing scientific guidance for online drug sales regulation and helping enhance the risk management capabilities of regulatory authorities. Despite these contributions, this study has some limitations.

First, because the extraction of risk nodes was performed manually, coding bias may exist in the process of translating the contents of cases of violation into risk nodes due to subjective judgment. To mitigate this potential bias, the following measures were taken: two researchers independently coded the cases, followed by cross‑checking and discussion; three experts were invited to review and validate the risk classification results; and final adjustments were made based on expert feedback. Nevertheless, completely eliminating coding bias remains difficult. Future research could use automated text analysis methods for cross‑validation to further reduce coding bias.

In addition, the online drug sales process involves numerous uncertain risks, and risk evolution patterns are relatively complex. Future research should continuously supplement violation case data and use multiple methods to deeply explore the patterns of risk propagation and key risk nodes in online drug sales, such as infectious disease models and node simulation attacks.

### Conclusion

As an emerging drug procurement method, online drug sales provide significant convenience to consumers, while also involving numerous risks. Based on the collected cases of violation, this study used complex network theory to construct a complex network model for safety risks in online drug sales. Using network indicators and the TOPSIS method, we measured the safety risks in online drug sales and identified key risks. The results showed that the key risk nodes in the complex network model include F15 (failure to strictly perform prescription review and dispensing duties), F01 (consumers failing to provide prescriptions), F02 (failure to issue prescriptions through internet hospitals), B12 (failure to establish drug procurement and acceptance records), and H22 (failure to establish drug sales records). Therefore, targeted risk prevention and control measures should be developed for these key risks to promote the healthy and orderly development of the online drug sales industry.

## Appendix

Multimedia Appendix 1 Steps in calculation used in the Technique for Order Preference by Similarity to Ideal Solution (TOPSIS) method.

Multimedia Appendix 2 Safety risks in online drug sales.

Multimedia Appendix 3 Ranking of C_i_ values for all nodes in the complex network model for safety risks in online drug sales.

## Abbreviations

TOPSIS: Technique for Order Preference by Similarity to Ideal Solution

## Data Availability

The datasets generated and analyzed during the study are available from the corresponding author upon reasonable request.
