# Supplementary material for: Measuring Safety Risks in Online Drug Sales: Empirical Study Based on Complex Network Theory
Source: JMIR Infodemiology. 2026 Jul 7;6:e86876. doi: 10.2196/86876 (PMC13389469; doi:10.2196/86876)
Supplement: Multimedia Appendix 1 [file infodemiology_v6i1e86876_app1.docx]

**Appendix 1. TOPSIS method calculation steps**

The calculation steps for the TOPSIS method are as follows:

(1) Construction of the Weighted Decision Matrix

Given m objects to be evaluated and n evaluation criteria, where x_ij_ represents the value of the j-th evaluation criterion for the i-th object, the initial decision matrix X is defined as:

$$\begin{aligned} X=\left[ \begin{matrix} x_{11} & \cdots& x_{1n} \\ \vdots& \ddots& \vdots\\ x_{m1} & \cdots& x_{mn} \end{matrix} \right]\#\left( 1 \right) \end{aligned}$$

Various evaluation metrics often employ different dimensions and units of measurement, with significant differences in magnitude. These metrics are categorized into positive-correlation risk indicators and negative-correlation risk indicators. For the former, higher metric values indicate greater risk, while for the latter, lower metric values signify higher risk. Therefore, to ensure the reliability of assessment results and eliminate the influence of dimensions on outcomes, data must undergo dimensionless conversion and trend normalization. For positive-correlation risk indicators, the standardized formula is:

$$\begin{aligned} y_{ij}=\frac{x_{ij}-(min)x_{ij}}{(max)x_{ij}-(min)x_{ij}}\#\left( 2 \right) \end{aligned}$$

For the negative-correlation risk indicator, the standardized formula is:

$$\begin{aligned} y_{ij}=\frac{(max)x_{ij}-x_{ij}}{(max)x_{ij}-(min)x_{ij}}\#\left( 3 \right) \end{aligned}$$

Then obtain the normalized decision matrix Y:

$$\begin{aligned} Y=\left( y_{ij} \right)_{m\times n}\#\left（ 4 \right） \end{aligned}$$

Subsequently, the entropy weight method was employed to determine the weights ω_j_ for each evaluation indicator. By combining the normalized decision matrix Y with the indicator weights ω_j_, the weighted normalized decision matrix Z was obtained:

$$\begin{aligned} Z=\left( \omega_{j}\times y_{ij} \right)_{m\times n}=\left( z_{ij} \right)_{m\times n}\#\left( 5 \right) \end{aligned}$$

(2) Determination of Positive and Negative Ideal Solutions

Positive and negative ideal solutions are derived from the weighted normative decision matrix Z. The positive ideal solution Z^+^ is defined by the maximum values of each indicator:

$$\begin{aligned} Z^{+}=\left\{ max Z_{ij}|i=1,2,3,\ldots,m \right\}=\left\{ Z_{1}^{+},Z_{2}^{+},\ldots,Z_{n}^{+} \right\}\#\left( 6 \right) \end{aligned}$$

The negative ideal solution Z^-^ is determined by the minimum values of each indicator:

$$\begin{aligned} Z^{-}=\left\{ min Z_{ij}|i=1,2,3,\ldots,m \right\}=\left\{ Z_{1}^{-},Z_{2}^{-},\ldots,Z_{n}^{-} \right\}\#\left( 7 \right) \end{aligned}$$

(3) Relative Nearness Degree Calculation

Based on Euclidean distance, calculate the distance from each evaluation object to the positive ideal solution and negative ideal solution, denoted as D+ i and D- i respectively:

$$\begin{aligned} D_{i}^{+}=\sqrt{\sum_{j=1}^{n} \left( Z_{j}^{+}-z_{ij} \right)^{2}}\left( i=1,2,\ldots,m \right)\#\left( 8 \right) \end{aligned}$$

$$\begin{aligned} D_{i}^{-}=\sqrt{\sum_{j=1}^{n} \left( z_{ij}-Z_{j}^{-} \right)^{2}}\left( i=1,2,\ldots,m \right)\#\left( 9 \right) \end{aligned}$$

Finally, calculate the relative nearness degree C_i_ for each evaluation target relative to the positive and negative ideal solutions, and determine their importance based on the magnitude of C_i_. The closer the relative nearness degree is to 1, the greater the impact of the evaluation target, necessitating prioritized prevention and control measures.

$$\begin{aligned} C_{i}=\frac{D_{i}^{-}}{D_{i}^{+}+D_{i}^{-}}\#\left( 10 \right) \end{aligned}$$
