# Supplementary material for: Measuring Safety Risks in Online Drug Sales: Empirical Study Based on Complex Network Theory
Source: JMIR Infodemiology. 2026 Jul 7;6:e86876. doi: 10.2196/86876 (PMC13389469; doi:10.2196/86876)
Supplement: Multimedia Appendix 2 [file infodemiology_v6i1e86876_app2.docx]

**Multimedia Appendix 2. Safety risks in online drug sales**

| **Category** | **Code** | **Risk** | **Definition** |
| --- | --- | --- | --- |
| Business qualifications | A01 | Failure to obtain a drug business license | Operating online drug sales without the legally required drug business license issued by the regulatory authority. |
|  | A02 | Failure to obtain a drug manufacturing license | Manufacturing drugs for online sale without a valid drug manufacturing license. |
|  | A03 | Failure to obtain the Online Drug Information Service Certificate | Providing online drug information services without the required certificate from the drug regulatory department. |
|  | A04 | Uploading a fake Drug Business License | Presenting a forged or altered Drug Business License on the online platform. |
|  | A05 | Operating beyond the business scope approved by the Drug Business License | Selling drug categories not listed in the approved business scope. |
|  | A06 | Operating beyond the business method permitted by the Drug Business License | Conducting drug business activities through methods or channels not authorized by the Drug Business License. |
|  | A07 | Failure to report to the drug regulatory department for engaging in online drug sales | Starting online drug sales without notifying the local drug regulatory department as required by law. |
|  | A08 | Failure to complete filing procedures with the public security organs | Operating an online drug sales website without completing mandatory cybersecurity or filing procedures with public security authorities. |
|  | A09 | Inability to provide the online drug sales filing certificate | Being unable to present the official filing certificate for online drug sales upon inspection. |
|  | A10 | Failure to obtain approval from the advertisement review authority | Publishing drug advertisements without prior approval from the designated advertisement review authority. |
|  | A11 | Using an expired Online Drug Information Service Certificate | Continuing to use the Online Drug Information Service Certificate after its validity period has lapsed. |
|  | A12 | Unapproved change of address on the Online Drug Information Service Certificate | Changing the registered business address on the certificate without obtaining regulatory approval. |
|  | A13 | Unauthorized change of online drug information service items | Adding, deleting, or modifying the types of drug information services provided without regulatory authorization. |
|  | A14 | Registering an online store using another person’s information | Using stolen or borrowed identity information to register an online drug store. |
|  | A15 | Lax review by the third-party platform | The online platform failing to adequately verify the qualifications of drug sellers operating on its site. |
|  | A16 | Lack of authorized permission | Engaging in any aspect of online drug sales without proper legal authorization from the competent authority. |
| Drug procurement | B01 | Procuring drugs from unqualified individuals or enterprises | Purchasing drugs from suppliers that do not hold the valid Drug Business License or manufacturing license. |
|  | B02 | Procuring imported drugs without drug approval documents | Buying imported drugs that lack approval numbers or registration certificates from the National Medical Products Administration (NMPA). |
|  | B03 | Procuring drugs without drug approval documents | Purchasing any drug (domestic or foreign) that does not have a valid drug approval document from the NMPA. |
|  | B04 | Purchasing medical institution preparations from medical institutions by queuing in person | Illegally obtaining hospital-made preparations (which are not for commercial sale) by hiring individuals to queue and buy them repeatedly. |
|  | B05 | Using others’ information to buy drugs from various hospitals | Using false or stolen identities to purchase prescription drugs from multiple hospitals. |
|  | B06 | Individuals purchasing prescription drugs in excessive quantities | A person buying more than the prescribed amount of a prescription drug. |
|  | B07 | Procuring counterfeit drugs | Knowingly or unknowingly purchasing counterfeit drugs (drugs with false ingredients or without active ingredients). |
|  | B08 | Procuring health insurance reflux drugs | Purchasing drugs that were originally dispensed under health insurance and then illegally resold (reflux drugs). |
|  | B09 | Procuring drugs obtained through criminal activities by others | Buying drugs that the seller acquired via theft, fraud, or other illegal means. |
|  | B10 | Failure to review the supplier’s qualification materials | Not checking or documenting the legal qualifications (licenses, GMP/GSP certificates) of the drug supplier. |
|  | B11 | Inability to provide drug procurement vouchers | Failing to produce original purchase invoices, receipts, or contracts proving the drug’s legitimate source. |
|  | B12 | Failure to establish drug procurement and acceptance records | Not keeping mandatory records of incoming drugs (e.g., batch number, quantity, supplier, acceptance date). |
|  | B13 | Illegal drug production | Producing drugs without legal authorization or in violation of applicable drug production regulations. |
|  | B14 | Illegal production of Chinese patent medicines | Producing traditional Chinese patent medicines without legal authorization or in violation of applicable drug production regulations. |
| Transportation and delivery | C01 | Failure to store and transport drugs according to specified conditions | Not maintaining required temperature, humidity, or light conditions during storage or delivery of drugs. |
|  | C02 | Temperature during packaging/transportation exceeding required storage temperature | The ambient temperature during courier packaging or transport rises above the drug’s safe limit. |
|  | C03 | Vehicle breakdown during cold chain drug distribution | Refrigeration vehicle or equipment failure during delivery of temperature‑sensitive drugs, risking drug degradation. |
|  | C04 | Failure to complete packing/sealing of refrigerated drugs in a cold environment | Packing or sealing refrigerated/frozen drugs at room temperature instead of in a controlled cold environment. |
|  | C05 | Failure to monitor and record temperature data in refrigerated containers in real‑time | Not having continuous temperature recording devices in cold chain packaging, or failing to keep logs. |
|  | C06 | Lack of cold chain transportation records during drug delivery | Failure to maintain required documentation of temperature-controlled conditions during the transportation of drugs requiring cold chain management. |
|  | C07 | Failure to sign a quality agreement with the entrusted delivery enterprise | The online drug seller not signing a formal agreement that defines quality responsibilities with the courier company. |
|  | C08 | Failure to supervise the entrusted delivery enterprise | Failure to oversee or monitor the compliance and performance of a contracted delivery enterprise in accordance with regulatory requirements. |
|  | C09 | Actual delivery situation inconsistent with the sales order | The drug type, quantity, or packaging received by the consumer differs from what was stated on the outbound delivery order. |
| Information display | D01 | Drug Business License information displayed on the website homepage is inconsistent with reality | The license number, business scope, or entity name shown online does not match the official license document. |
|  | D02 | Failure to promptly update and publicize Drug Business License information | Continuing to display outdated license information after renewal, change of address, or other modifications. |
|  | D03 | Failure to display Drug Business License information prominently on the website homepage | Hiding the license information or placing it in a hard‑to‑find location instead of the homepage’s prominent area. |
|  | D04 | Failure to display qualification information of pharmacists or other pharmaceutical technical personnel | Not showing the names, certificates, or license numbers of the responsible pharmacists on the website. |
|  | D05 | Failure to mark the certificate number of the Online Drug Information Service Certificate prominently on the website homepage | Omitting or obscuring the required certificate number of the Online Drug Information Service Certificate. |
|  | D06 | Displaying prescription drugs as OTC | Presenting prescription drugs as over-the-counter (OTC) products without requiring a prescription. |
|  | D07 | Drug information display page is inconsistent with reality | Product information (e.g., manufacturer, approval number, ingredients) on the webpage differs from the actual drug. |
|  | D08 | Failure to highlight prescription drug risk warning information on the drug display page | Not posting a clear warning message such as “Prescription drug – use only under physician guidance” on the product page. |
|  | D09 | Failure to fully inform consumers of relevant risk warnings in promotional content | Omitting side effects, contraindications, or other safety information in the advertisement or promotional text. |
|  | D10 | Using others’ trademarks for commercial identification and misleading references | Unauthorized use of another company’s trademark to imply endorsement or confuse consumers. |
|  | D11 | Directly displaying the packaging and labels of prescription drugs | Making the packaging and labels of prescription drugs visibly accessible to consumers without appropriate controls or professional oversight. |
| Advertising and promotion | E01 | Purchasing followers to spectate livestreams | Artificially inflating viewership of drug promotion livestreams by buying fake followers or bots. |
|  | E02 | Modifying online store sales volume | Falsifying sales numbers (e.g., using fake orders) to make a drug appear more popular than it actually is. |
|  | E03 | Infringing on others’ distribution rights on online platforms | Selling drugs through unauthorized channels or interfering with the exclusive distribution rights granted to others. |
|  | E04 | False drug promotion through fictitious transactions | Creating fake transactions and positive reviews to mislead consumers about drug efficacy or popularity. |
|  | E05 | Fabricating and spreading price increase information | Inventing news of an impending drug price hike to induce panic buying or boost sales. |
|  | E06 | Inability to provide basis for promotional content | Failing to present scientific evidence or official data supporting claims made in drug promotions. |
|  | E07 | Drug advertisements containing assertions or guarantees about efficacy or safety | Using language such as “100% effective” or “absolutely safe”, which is prohibited for drug ads. |
|  | E08 | Advertisements containing comparisons with the efficacy and safety of other drugs | Directly comparing a drug’s performance to that of another brand or generic drug. |
|  | E09 | Advertised drug efficacy inconsistent with the approved instructions | Promoting a drug for a disease or symptom not listed in its officially approved drug label. |
|  | E10 | Publishing prescription drug advertisements outside designated professional publications | Running ads for prescription drugs in mass media (TV, outdoor billboards, general websites) instead of only in medical journals. |
|  | E11 | Publishing promotional content containing well-known trademarks | Using famous trademarks not owned by the seller to imply superior quality or endorsement. |
|  | E12 | Publishing drug advertisements containing false or misleading content | Making any untrue statement about the drug, its effects, or its origin that could deceive consumers. |
|  | E13 | Advertisement content inconsistent with the Advertisement Review Approval Decision | Running an ad that differs from the version approved by the advertisement review authority. |
|  | E14 | Advertisement content contains efficacy or cure rate language | Advertising content that includes claims about drug efficacy or specific cure rates, which may mislead consumers regarding treatment outcomes. |
|  | E15 | Failure to inspect the Drug Advertisement Review Certificate | Publishing a drug advertisement without checking whether the advertiser has a valid review certificate. |
|  | E16 | Promoting drugs online as patented without obtained patent authorization | Claiming patent protection for a drug when no patent has been granted or the patent has expired. |
|  | E17 | Counterfeit patent promotion | Promoting a product by falsely claiming it is protected by a patent or by misrepresenting patent status or scope. |
| Prescription management | F01 | Consumers failing to provide prescriptions | The buyer does not upload or present a valid prescription before purchasing a prescription drug. |
|  | F02 | Failure to issue prescriptions through internet hospitals | The online pharmacy dispenses a prescription drug based on a prescription that was not generated by a licensed internet hospital. |
|  | F03 | Consumers provide invalid prescriptions | The uploaded prescription is expired, incomplete, or written for a different patient/drug. |
|  | F04 | Uploading a blank prescription form | Submitting a prescription form with no doctor’s signature, diagnosis, or drug information. |
|  | F05 | Failure to implement the real-name system as required | Not verifying the consumer’s real identity (name, ID number) before selling prescription drugs. |
|  | F06 | Failure to verify patient information | Not checking whether the prescription corresponds to the actual patient’s name, age, and medical history. |
|  | F07 | Failure to verify prescription information | The online pharmacy does not check the prescription’s authenticity, dosage, or compatibility. |
|  | F08 | Prescribed user on the e‑prescription differs from the actual user | The person named on the prescription is different from the person who will use the drug. |
|  | F09 | Drugs on the prescription form differ from the actual purchased drugs | A discrepancy in which the drugs listed on a prescription differ from those actually dispensed to or purchased by the patient. |
|  | F10 | Using a departed employee’s account to log into the online drug sales platform | An active employee uses the login credentials of a former pharmacist or reviewer to process prescriptions. |
|  | F11 | Non‑pharmacist or pharmaceutical technical personnel reviewing prescriptions | Allowing a person without a pharmacist license or pharmaceutical technician certification to perform prescription review. |
|  | F12 | Pharmacist failed to review, confirm, and sign on the e‑prescription | The prescription is dispensed without being reviewed and signed off by a licensed pharmacist. |
|  | F13 | Prescription reviewer’s signature is not authentic | The signature of the prescription reviewer is forged or not made by the authorized individual. |
|  | F14 | Failure to pass prescription review | The prescription review process is skipped or the system allows dispensing despite failing the review. |
|  | F15 | Failure to strictly perform prescription review and dispensing duties | The pharmacist or system does not thoroughly check drug interactions, contraindications, or appropriate dosage before dispensing. |
|  | F16 | Selling prescription drugs without a prescription | Completing a sale of a prescription drug without the buyer providing any valid prescription. |
|  | F17 | E‑prescription not marked after use | An electronic prescription that is not marked as used or consumed after dispensing, potentially allowing repeated or unauthorized reuse. |
|  | F18 | Failure to retain prescriptions as required | Not keeping copies of paper or electronic prescriptions for the legally mandated retention period. |
|  | F19 | Failure to sign an agreement with the e‑prescription provider | The online pharmacy has no formal contract with the internet hospital or prescription system provider. |
| Pharmaceutical services | G01 | Lack of an online pharmaceutical service system | No interactive system (chat, phone, or video) for consumers to ask pharmacists about drug safety before purchase. |
|  | G02 | Failure to employ licensed pharmacists or other legally qualified pharmaceutical technical personnel | Failure to hire licensed pharmacists or other legally qualified pharmaceutical technical staff required by law. |
|  | G03 | Failure to inform the purchaser of drug usage, dosage, and precautions during sale | The seller does not provide clear instructions or warnings (e.g., side effects, storage) at the time of sale. |
| Drug sales | H01 | Bundling and tying drug sales in combination sets | Forcing consumers to buy a set that includes a less‑popular drug together with the one they want. |
|  | H02 | Adopting methods of tying sales or buying drugs to get free drugs | Adopting sales tactics that require purchasing one drug to obtain another (tying sales) or offering free drugs as a promotional incentive. |
|  | H03 | Giving prescription drugs or Class A OTC drugs to the public | Offering free samples or giveaways of prescription drugs or Class A OTC drugs (those requiring pharmacist supervision). |
|  | H04 | Disguisedly increasing drug prices | Increasing the price by reducing package size, changing dosage form, or adding hidden fees, while claiming no price increase. |
|  | H05 | Illegal drug sales | Any sale of drugs that violates the Drug Administration Law or online drug sales regulations. |
|  | H06 | Illegal sales of medical institution preparations | Selling hospital‑made preparations (which are only for internal use) to the general public online. |
|  | H07 | Illegal sales of Chinese patent medicines / Chinese herbal pieces | Selling traditional Chinese medicine products without complying with registration, labeling, or licensing requirements. |
|  | H08 | Illegal sales of over-the-counter (OTC) drugs | Selling OTC drugs in violation of applicable laws or regulatory requirements. |
|  | H09 | Illegal sales of drugs beyond the approved business scope | Selling drugs that fall outside the seller’s permitted drug categories. |
|  | H10 | Illegal retail drug sales | An entity without a retail license selling drugs directly to consumers online. |
|  | H11 | Illegal sales of health insurance reflux drugs | Selling drugs that were originally obtained through health insurance reimbursement and then illegally resold. |
|  | H12 | Conducting livestreams with free drug giveaways | Using livestream platforms to offer free drugs as giveaways, which may violate drug promotion and distribution regulations. |
|  | H13 | Selling drugs prohibited from online sales | Selling drugs that are explicitly banned from internet sales by the NMPA. |
|  | H14 | Selling imported drugs without drug approval documents | Offering imported drugs that have not been registered or approved by the NMPA for sale in China. |
|  | H15 | Selling drugs without drug approval documents | Selling any drug that has no valid NMPA approval number (unregistered domestic or foreign drug). |
|  | H16 | Selling drugs under special state control | Selling controlled substances such as narcotics, psychotropic drugs, or drug precursors without special authorization. |
|  | H17 | Selling medicinal toxic drugs | Selling pharmaceutical products classified as medicinal toxic drugs without proper authorization or in violation of regulatory requirements. |
|  | H18 | Selling drugs obtained through criminal activities by others | Selling drugs that the seller knows or should know were stolen, fraudulently obtained, or illegally imported. |
|  | H19 | Illegal sales of counterfeit drugs | Selling counterfeit drugs in violation of applicable drug laws. |
|  | H20 | Drug sales not registered in the computer system | Failing to record the sale in the mandatory digital traceability system. |
|  | H21 | Failure to issue drug sales vouchers | Not providing the consumer with a sales receipt or invoice showing the drug name, quantity, and date of purchase. |
|  | H22 | Failure to establish drug sales records | Keeping no records of which drugs were sold, to whom, and in what quantity. |
|  | H23 | Drug sales records are not authentic or complete | The sales records are deliberately falsified or missing key information. |
|  | H24 | Sales voucher content is incomplete | The sales receipt omits required information such as the seller’s name, license number, or drug specification. |
|  | H25 | Illegal provision of online drug information services | Providing drug-related information services via the internet without the required authorization or in violation of applicable regulations. |
|  | H26 | Illegal provision of online drug information services at a new address | Moving the drug information service to a new domain or physical server location without re‑registration. |
|  | H27 | Failure to retain network logs as required | Not storing website visit logs, transaction records, or IP addresses for the period mandated by law. |
