# Supplementary material for: Measuring Safety Risks in Online Drug Sales: Empirical Study Based on Complex Network Theory
Source: JMIR Infodemiology. 2026 Jul 7;6:e86876. doi: 10.2196/86876 (PMC13389469; doi:10.2196/86876)
Supplement: Multimedia Appendix 3 [file infodemiology_v6i1e86876_app3.docx]

**Multimedia Appendix 3. Ranking of Ci values for nodes in the complex network model for safety risks in online drug sales.**

| **Node** | **C_i_ in descending order** |
| --- | --- |
| F15 | 0.5408 |
| F01 | 0.4638 |
| F02 | 0.4419 |
| B12 | 0.3956 |
| H22 | 0.3872 |
| F19 | 0.3561 |
| H19 | 0.3547 |
| B07 | 0.3518 |
| E14 | 0.3477 |
| F09 | 0.3477 |
| F13 | 0.3477 |
| B05 | 0.3471 |
| D04 | 0.3471 |
| D07 | 0.3471 |
| E04 | 0.3471 |
| H05 | 0.3041 |
| B11 | 0.3008 |
| A01 | 0.2830 |
| B03 | 0.2822 |
| H08 | 0.2812 |
| B01 | 0.2795 |
| B10 | 0.2618 |
| F16 | 0.2528 |
| D08 | 0.2509 |
| E09 | 0.2049 |
| B02 | 0.1814 |
| H15 | 0.1750 |
| E07 | 0.1719 |
| H06 | 0.1667 |
| F07 | 0.1631 |
| C01 | 0.1531 |
| E10 | 0.1520 |
| H14 | 0.1518 |
| A10 | 0.1504 |
| A14 | 0.1485 |
| D06 | 0.1481 |
| D09 | 0.1148 |
| F05 | 0.1148 |
| H16 | 0.1124 |
| H23 | 0.1070 |
| D03 | 0.1015 |
| H13 | 0.0981 |
| E12 | 0.0972 |
| F18 | 0.0920 |
| A09 | 0.0754 |
| F17 | 0.0729 |
| H17 | 0.0716 |
| H20 | 0.0716 |
| H10 | 0.0628 |
| H04 | 0.0421 |
| H25 | 0.0419 |
| E06 | 0.0383 |
| F08 | 0.0382 |
| A04 | 0.0262 |
| B08 | 0.0214 |
| B09 | 0.0214 |
| D05 | 0.0214 |
| D11 | 0.0214 |
| E02 | 0.0214 |
| G03 | 0.021 |
| A16 | 0.0204 |
| E13 | 0.0204 |
| A02 | 0.0204 |
| E11 | 0.0065 |
| H11 | 0.0065 |
| H18 | 0.0065 |
| B13 | 0.0026 |
| B14 | 0.0026 |
| C04 | 0.0026 |
| C06 | 0.0026 |
| C08 | 0.0026 |
| D02 | 0.0026 |
| D10 | 0.0026 |
| E03 | 0.0026 |
| E08 | 0.0026 |
| E17 | 0.0026 |
| H03 | 0.0026 |
| H07 | 0.0026 |
| H09 | 0.0026 |
| H12 | 0.0026 |
| H26 | 0.0026 |
| H27 | 0.0026 |
